# Mecoptera of Canada

**DOI:** 10.3897/zookeys.819.26627

**Published:** 2019-01-24

**Authors:** David C.A. Blades

**Affiliations:** 1 Research Associate, Royal British Columbia Museum, 675 Belleville St, Victoria, BC, V8W 9W2, Canada Royal British Columbia Museum Victoria Canada

**Keywords:** biodiversity assessment, Biota of Canada, Mecoptera, scorpionfly

## Abstract

The Mecoptera are represented in Canada by 25 extant species in four families, an increase of three species since the prior assessment in 1979. An additional 18 or more species and one family are expected to occur in Canada based on distributional records, recent collections and DNA analyses. The Barcode of Life Data System currently lists 24 Barcode Index Numbers for Canadian Mecoptera. There are nine species of fossil Mecoptera known from Canada.

Mecoptera, commonly known as scorpionflies or hangingflies, is one of the smaller insect orders with about 600 extant species in nine families worldwide (Penny 1997). The order is represented in the fossil record dating back to the Permian about 200 million years ago (Webb et al. 1975). The paleobiology database (https://fossilworks.org) currently lists a total of 686 species in 40 families globally, 30 species from North America and nine of those found in Canada. Several early Eocene fossil species were recently discovered in British Columbia (Archibald 2005, 2009, 2010, Archibald et al. 2013).

The extant fauna of Canada and the USA consists of 87 species in five families (Penny 1997, Blades 2016) with 25 species currently known from Canada (Table 1). The greatest diversity of Mecoptera in Canada occurs in the Mixedwood Plains ecozone of southern Ontario, totaling 19 species in four families. Some of those species range westward to southeastern Manitoba and eastward to the Maritimes (Cheung et al. 2006). Boreidae (snow scorpionflies) is the only family represented from the Rocky Mountains westward in Canada. The family Panorpodidae, which includes two species, *Brachypanorpasacajawea* Byers and *B.oregonensis* (MacLachlan), found in the bordering states of Washington, Idaho and Montana, may also occur in southern British Columbia (Byers 1997).

**Table 1.** Census of Mecoptera in Canada.

Taxon^1^	No. species reported in Downes (1979)	No. species currently known from Canada	No. BINs^2^ available for Canadian species	Est. no. undescribed or unrecorded species in Canada	General distribution by ecozone^3^	Information sources
Boreidae	7	9^4^	6	>4	Montane Cordillera, Pacific Maritime, Western Interior Basin, Mixedwood Plains, Atlantic Maritime	Penny 1997, GBIF 2017
Panorpidae	10	12	16	11	Mixedwood Plains, Atlantic Maritime, Boreal Shield, Prairies	Penny 1997, Cheung et al. 2006, GBIF 2017
Bittacidae	4	3	1	1	Mixedwood Plains, Prairies	Penny 1997, Cheung et al. 2006, GBIF 2017
Meropeidae	1	1	1	0	Mixedwood Plains	Penny 1997, Cheung et al. 2006, GBIF 2017
Panorpodidae	0	0	0	2	Pacific Maritime, Montane Cordillera	Penny 1997, GBIF 2017
**Total**	**22**	**25**	**24**	**>18**		

**^1^**Classification follows that indicated in Downes (1979). **^2^**Barcode Index Number, as defined in Ratnasingham and Hebert (2013). ^3^See figure 1 in Langor (2019) for a map of ecozones. **^4^**Count of known Boreidae includes one species of *Caurinus* as this genus has been collected at several locations in coastal British Columbia (DCA Blades and C Wood unpubl. data).

The number of extant species known from Canada has increased by three (14%) since the previous assessment by Downes (1979). One new species of Boreidae has been described and at least four more species are expected to occur in British Columbia (Blades 2002, Canadian Endangered Species Conservation Council 2016; DCA Blades and C Wood unpubl. data). Other changes in the known fauna include the addition of two Panorpidae species and the removal of one Bittacidae species that was included in the 1979 assessment. There are no known endemic or non-native species in the Canadian fauna.

The Mecoptera of Canada are relatively well known compared with many insect orders. Undescribed extant species for Canada are expected for Boreidae and Panorpidae. Barcode Index Numbers (BINs) for *Panorpa* suggest that a number of species are likely to be species complexes. Areas of research on Canadian Mecoptera that are currently lacking include basic biology, such as the life histories, biogeography and species in the fossil record.
